# Vaginal Obliteration

**Published:** 1838

**Authors:** Thomas Carrall

**Affiliations:** St. Clairsville, Ohio


### Art. III.—Vaginal Obliteration.

*Two cases of Obliteration of the Vagina, caused by ulceration.* By Thomas Carrall, M. D., of St. Clairsville, Ohio.

*Case* 1.—I was requested, sometime during the spring of 1835, to visit a lady, for the purpose of assisting Dr. H—— in performing an operation on her. I attended, according to request, and found that the lady had been delivered of a still-born child, some months before; that the labor had been difficult; that the child's head had rested in the inferior strait for many hours; and that soon after delivery, ulceration of the vagina had taken place, and had terminated in the obliteration of a considerable portion of it. Not long after her recovery, the usual symptoms of menstruation were felt, and they had returned every month until the time I saw her; but without any external discharge. She complained of swelling in the lower part of the abdomen, and pain in the back.

From this most disagreeable situation it was thought she might be relieved, by making an artificial passage, which might be prevented from closing, by keeping the wound filled with lint, or some other substance. Upon examination, we found the vagina pervious for about three-fourths of an inch above the meatus urinarius. It seemed obvious, that the operation would be difficult and dangerous; and that the soft parts below the adhesion must be in great danger of injury, unless protected in some way from the edge of the knife. But Dr. H. had no instrument that would give the necessary protection. I advised him to defer the operation for a few days, and to obtain three crooked spatulas, by which the mucous membrane could be defended. He agreed to this proposition, and brought the instruments to our next meeting, which took place in a few days. We fixed one of the spatulas so that the meatus was protected, and one on each side; the handles being completely out of the way, owing to the bend in each. The Doctor now commenced the operation with a scalpel, which was by no means sharp; but cut and tore his way in the direction of the axis of the vagina, until I could introduce my index finger to the second joint. The opening was made in an oblique direction upwards, and to the left side of the vagina. No entry was made into the canal above; but the operator desisted from further efforts. Lint was stuffed into the opening, and the patient removed to bed.

During the next day, however, a large quantity of grumous blood and serum, passed througb the opening, which proved that a communication had been effected by the pressure of the fluid contained in the uterus. On examination, we found that a sinuous opening was formed, and through this the regular menstrual evacuations continued to pass, at each period, for a few months, when the lady again became pregnant. About four or five months after impregnation, abortion took place. The fœtus, enveloped in the membranes, was ejected from the womb, and pushed forcibly against the point of obstruction; which at length gave way.

About a year after this occurrence, I was summoned to wait on this same lady, who was then in labor. The fœtus had attained to the full period of maturity, and was unusually large. I found, on examination, that there was a ligamentous band surrounding the vagina, about an inch and a quarter wide below and laterally, but somewhat narrower above; and at this point it was pressed down a little, like a flap, which became still narrower, or thinner, as the child advanced. The labor was peculiarly difficult; for the ligamentous state of the vagina resisted, for a number of hours, all the efforts of the uterus to press the head through it. However, it was gradually dilated, and I became convinced that the child would be born without the use of instruments, and probably alive. As to the latter I was, however, deceived; for just as the labor seemed to be almost completed, a sudden cessation of pain took place, and the patient fell into a condition bordering on convulsions, in which she continued too long for the safety of the child. In the meantime I prepared to use the forceps; but fortunately the child's head was expelled by a single pan. As this woman is young, and possesses a fine constitution, I have the flattering hope that she will yet have a living child; for I think the obstruction will be less with every succeeding labor.

*Case* 2.—A woman of robust constitution had difficult labor. The head of the child rested against the perinœum for about 24 hours; but the pains eventually effected the expulsion of the child without the use of instruments. No untoward symptom occurred for some days after delivery; but this did not continue, for inflammation of the mucous membrane of the vagina set in, and continued until it terminated in the obliteration of that passage. This result was unfortunately not suspected by the friends of the patient, nor by her physician, who lived at some distance, and who had not been sufficiently apprized of the nature of the case. Some months after this period, her physician was consulted by her husband, and informed that a complete obliteration of the natural passage had taken place. The patient was advised to apply to the writer of this article. She accordingly called on me; and upon examination, I found, apparently, a complete obliteration of the vaginal canal, which commenced about three-fourths of an inch above the meatus urinarius.

The patient, however, had menstruated regularly for some months; but said that much difficulty and pain occurred before the discharge became visible; and when it did, there was considerable fluid evacuated for some hours. I asked her whether she thought it passed through the urethra or not; she said she was confident that it did. With this opinion I could not agree. I imagined that there might be a sinous opening, which closed between the menstrual periods, and that the pressure of the menstrual fluid re-opened it. As this seemed to me to be the only way the matter could be accounted for, I directed the patient to go home, and to send for me when the menstrual discharge should be again present.

Within about two weeks I was requested to see her; and, upon examination, found my anticipations correct. A very small opening had been effected by the retained fluid. I introduced a probe, and found it went the whole length of the vagina. It was agreed, upon consultation with her physician, Dr. J. Alexander, that a bougie of the size of a common catheter, should be introduced immediately, and retained in the sinus for one or two days; and that it should then be withdrawn, and a larger one put in its place; and that a still larger one should be used at the end of a few days; and that an increased size should be continued until the vagina should be restored to its natural dimensions. Dr. A. agreed to make instruments of linen and wax, rolled into cylinders, and polished; and he succeeded to admiration; for in about two months our object was accomplished.

I was called, on the 7th July, 1836, to see this lady, who was in labor. This was thirteen months from the period when the bougies were dispensed with six or eight hours before, and had been severe, as I was informed, by Dr. A., who had been with her most of the time. It was found, upon examination, that the part of the vagina which had been dilated was very rigid and unyielding; that it commenced about three-fourths of an inch above the meatus, and was about one inch in length.

We concluded to let the labor progress for three or four hours longer, and see what would be the effect. We however agreed to give twenty grains of ergot, which quickened the pains very much; but notwithstanding this, we had the mortification to find, that very little was done in two hours towards dilating the legimentus band. The stricture, however, appeared to be pressed down as low as the meatus, and formed a flap above, with a circular edge, that was quite thin, and about three-fourths of an inch long, or wide, from the meatus. The band was the narrowest on each side. We now decided on an operation, and concluded to cut nearly through the band on the right side; which was, however, but in part effected; for I was forced to desist, in consequence of profuse hæmorrhage. During every pain the wound bled very freely; the head of the child seemed to act as a ligature on the veins; a pint and a half of blood was soon lost. I caught the flap above, and forcibly lacerated it with my finger, and with some exertion introduced the female leg of the forceps, and succeeded in a few seconds to fix both legs on the head; I then drew the head against the stricture with considerable force. The parts resisted with much obstinacy. I however succeeded in effecting the delivery, in ten or fifteen minutes. The bleeding ceased; but the child was still-born. The woman did well, although suppuration was profuse. She rode ten miles in one day, three or four weeks after delivery.

This woman has been again confined within the last month or two, and would in all probability have had a living child, had it been healthy; but it was affected with watery head; which made it necessary to again use the forceps, although the breech first presented. Her physician, Dr. Alexander, informs me, that she is again up and doing well. He says, that the ligamentus band around the vagina offered but little resistance, and thinks that if a head of the natural size had presented, but little difficulty would have been experienced in effecting a safe delivery. I think that this woman will yet have a living child, should she continue to have her health. It was suspected that there was deformity of the pelvis in her case. I however think that it is not so, or but in a very slight degree.

The foregoing case has admonished me, that in all cases where obliteration of the vaginal canal, or part of it, has taken place; (for I think the whole canal never becomes obliterated,) a very narrow opening through the obstruction is sufficient; say that it would admit the index finger; or if it were even less, so that it communicated with the canal above, I think it would be sufficient. Nothing would then remain to be done, but to dilate the artificial canal by bougies.

Where an operation of the kind under consideration is to be performed, the operator should be sure that he understands the anatomy of the parts, and that he has a steady hand. When we reflect how very nearly the bladder and rectum approximate, when there is an alteration of the vagina, we shall at once see the great danger of wounding one or both of these organs. To avoid wounding them, the operator should introduce the index finger of the left hand into the rectum, and use it as a director for the knife. By this precaution, I think the danger of wounding the bladder or rectum would be much diminished. In performing an operation of so delicate a nature, time and patience are necessary.

It will be recollected, that in one of these cases impregnation was effected, when but a sinuous vaginal opening existed; but that in both of them there was an actual communication with the uterus.

January 1838.

